# What Is Your Diagnosis? Globular to amorphous material in peripheral blood smears

**DOI:** 10.1111/vcp.70008

**Published:** 2025-06-03

**Authors:** Em Adam, Craig Thompson, Andrea Pires dos Santos, Joanne Messick

**Affiliations:** ^1^ Purdue University West Lafayette Indiana USA; ^2^ Purdue Institute for Cancer Research West Lafayette Indiana USA

## 1 CASE PRESENTATION

Three 8‐week‐old research dogs (two male intact Victorian Bulldogs and one female intact Shih Tzu) had peripheral blood submitted to the Purdue University Veterinary Hospital Clinical Pathology Laboratory (PUVHCPL) for a health screening. These dogs were enrolled in a study aimed at understanding the impact of ground transportation on puppy welfare. All research dogs were considered healthy and had no significant abnormalities observed on physical exam. The dogs were not currently being injected with any fluids or medications. However, as a form of pain management during blood draws, lidocaine cream was applied locally at the blood draw site five minutes before blood collection on each dog. The blood draw site was performed with a 22‐, 23‐, or 25‐gauge needle and included either the jugular vein or cephalic vein. The medical technologist at PUVHCPL observed an unknown material observed at the feathered edge of all blood smears from these dogs, which triggered a review by the clinical pathologist for further evaluation.

## 2 PERIPHERAL BLOOD INTERPRETATION: AMORPHOUS TO GLOBULAR, PURPLE TO MAGENTA MATERIAL CONSISTENT WITH LIDOCAINE CREAM

The peripheral blood of one Victorian Bulldog was analyzed using the Advia 2120i Hematology System (Siemens, Washington, DC). Additionally, all three research dogs had peripheral blood smears stained with Modified Wright stain and processed using the Siemens Hema‐Tek 3000 system. Since the other two research dogs were only submitted for slide review, their blood was not analyzed by the Advia system. The scatterplots from the Victorian Bulldog only showed abnormalities in the “Baso” channel (Figure 1), where a continuous, linear abnormality is seen going through the mononuclear area of the scatterplot and into where basophils are typically located. This abnormality is presumed to be due to the lidocaine gel since no other abnormalities that could cause this change were noted in the dog's peripheral blood. The smears were evaluated by a clinical pathology resident (EA) and deemed to be of good diagnostic quality without evidence of any age‐related changes. Leukocyte numbers and erythrocyte density of two dogs were within normal limits. One dog had minimal macrocytic hypochromic anemia. A few reactive lymphocytes, as well as a moderate amount of poikilocytes, rare polychromatophils, clumped platelets, and rare large platelets, were observed on all of the blood smears. Despite clumping and mild size variability, a manual estimate of platelet numbers was within normal limits. In addition, low to moderate amounts of amorphous to globular, purple to magenta, extracellular material was observed at the feathered edge and occasionally in the monolayer of the peripheral blood smears. Differentials for this material included an artifact of sampling or staining precipitate, a proteinaceous or mucinous material, or material associated with the use of lidocaine cream (Uber Numb 5% Lidocaine Numbing Cream, UberScientific, LLC, Lexington, KT)^1^ as part of pain management during blood draws. To verify that this material was associated with the use of lidocaine cream, the researchers provided the cream for further investigation.

**FIGURE 1:**
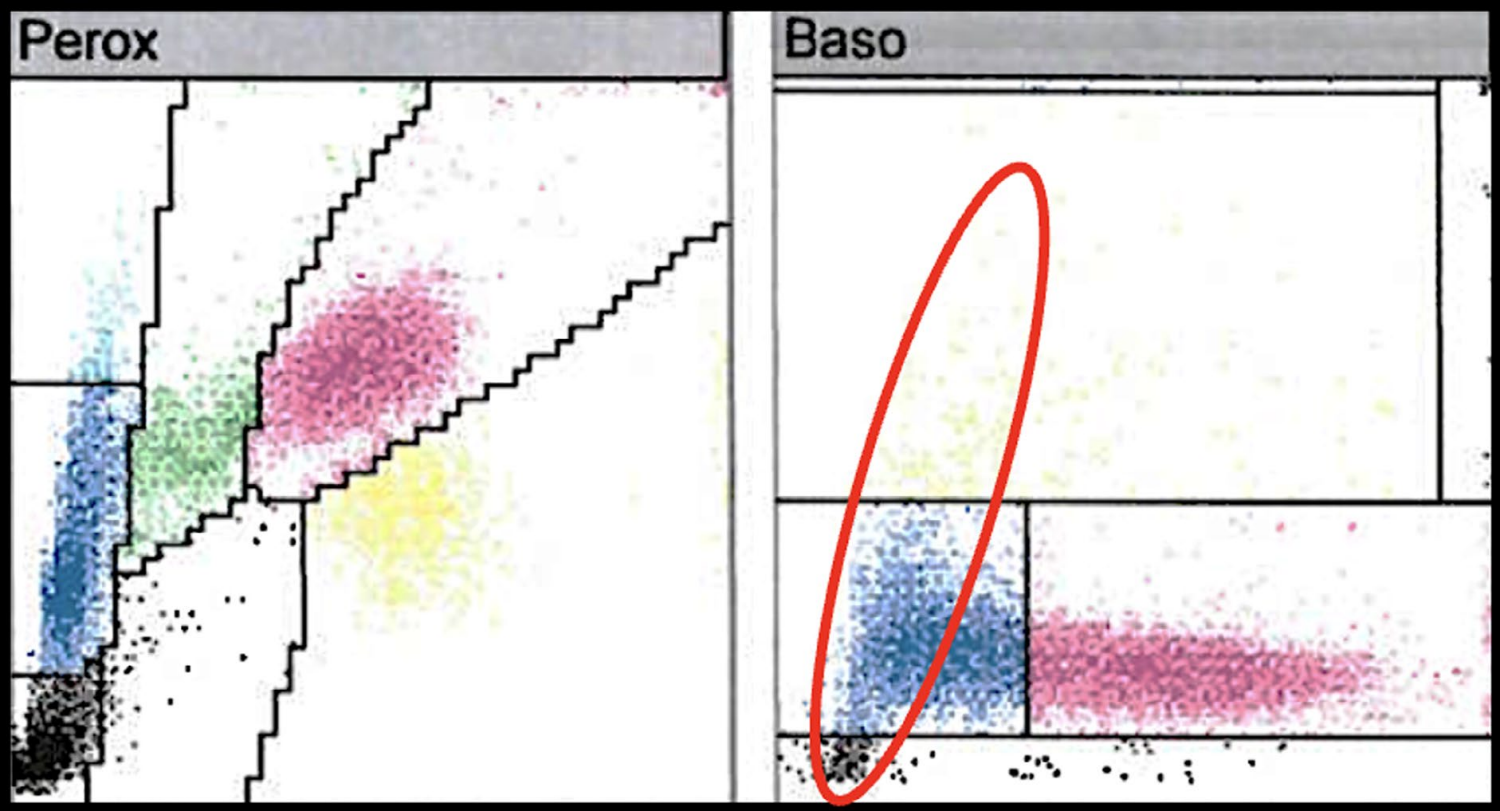
Scatterplot data from Advia 2120i Hematology system from one of the research dogs, a male intact Victorian Bulldog. While the “Perox” channel has no overt abnormalities, the “Baso” channel has a continuous, linear abnormality seen (red circle) going through the mononuclear area (blue) of the scatterplot and into where basophils (yellow) are typically located. This abnormality is presumed to be due to the lidocaine gel since no other abnormalities that could cause this change were noted in this dog's peripheral blood.

Whole blood in 3.0‐mL K2‐EDTA tubes from one dog and horse were obtained from excess samples. These samples were mixed gently and slowly warmed to ambient temperature. Four to five drops of each blood sample (dog and horse) were placed into two separate test tubes, and a small amount of the lidocaine cream (less than 0.5 g) was gently mixed with the blood. Another test tube not containing lidocaine cream was used as a control for each species. Peripheral blood smears were made from each test tube, stained with Modified Wright stain using the Siemens Hema‐Tek 3000, and evaluated by the same clinical pathology resident (EA).

As seen in Figures 2A and 3A, no globular to amorphous material is seen at the feathered edge. However, in Figures 2B and 3B, where the lidocaine cream was added to the peripheral blood, abundant amounts of globular to amorphous, purple to magenta material can be seen at the feathered edge. This material was morphologically indistinguishable from the unknown material identified on the blood smears from the research animals (Figure 4). Therefore, we concluded that the material at the feathered edge of the blood smears from these research dogs was consistent with the lidocaine cream used for pain management.

**FIGURE 2:**
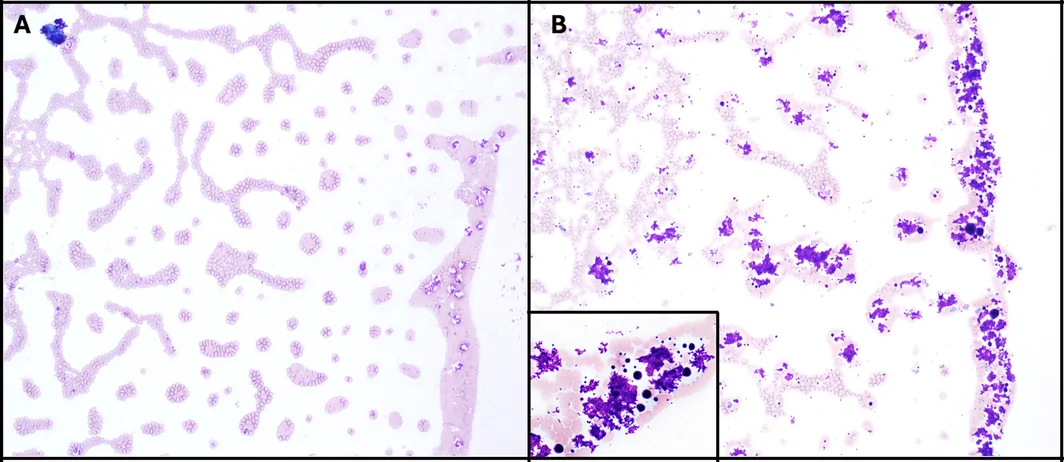
Photomicrograph of peripheral blood in a dog. (A) Peripheral blood without lidocaine cream added at 10× objective. (B) Peripheral blood after the lidocaine cream is added, showing abundant amounts of amorphous to globular, purple to magenta material at the feathered edge at 10× objective. Inset: 60× objective. Modified Wright stain.

**FIGURE 3:**
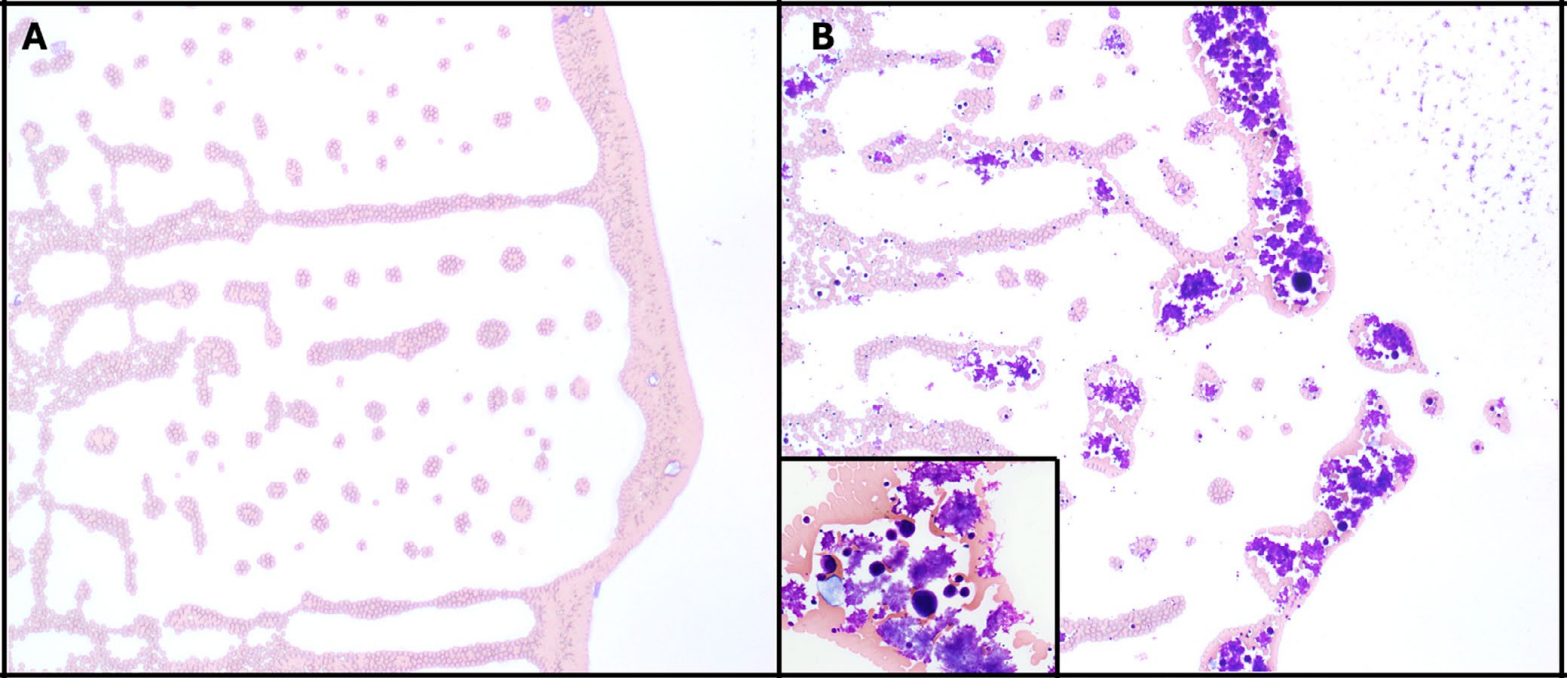
Photomicrograph of peripheral blood in a horse. (A) Peripheral blood without lidocaine cream added at 10× objective. (B) Peripheral blood after the lidocaine cream is added, showing abundant amounts of amorphous to globular, purple to magenta material at the feathered edge at 10× objective. Inset: 60× objective. Modified Wright stain.

**FIGURE 4:**
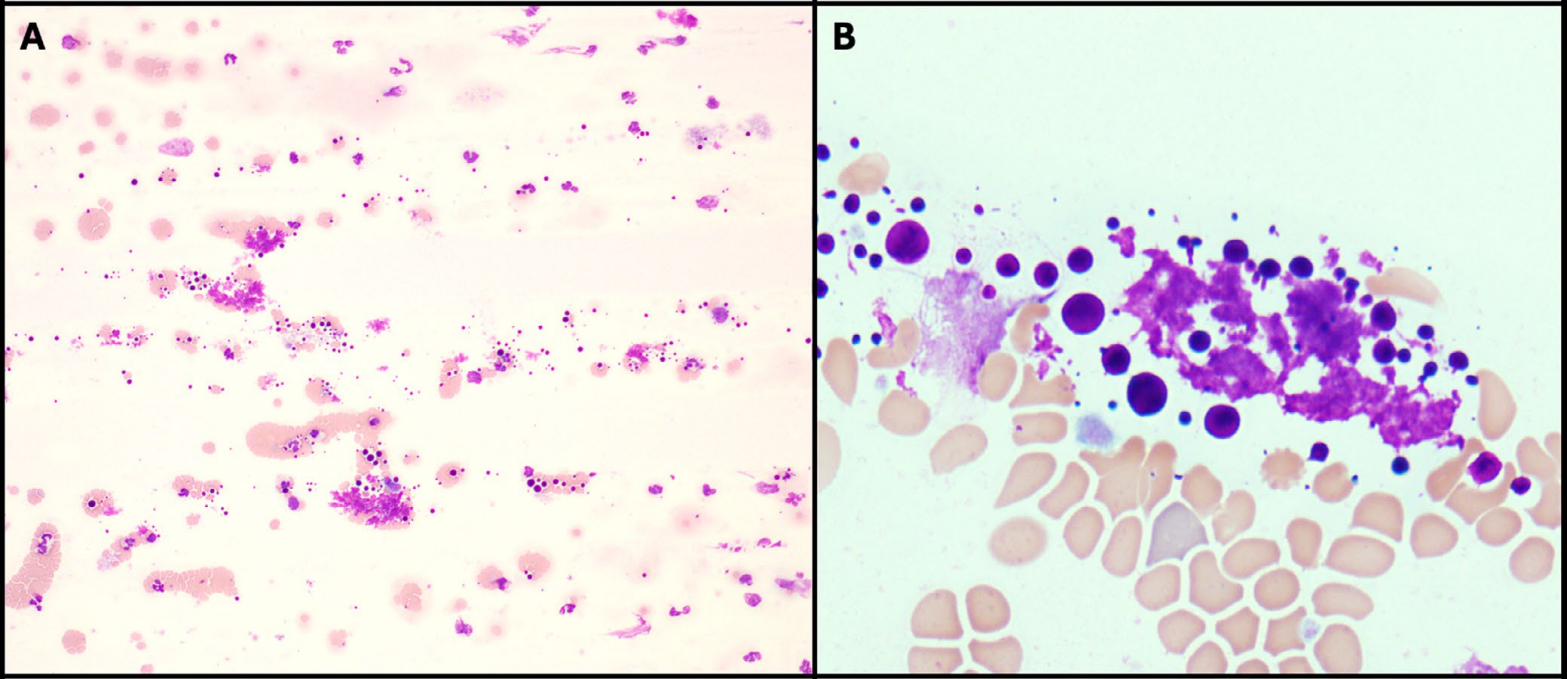
Photomicrograph of peripheral blood in a research dog. Modified Wright stain, (A) 10× objective, (B) 60× objective.

## 3 DISCUSSION

Lidocaine cream Uber Numb 5% Lidocaine Numbing Cream, UberScientific, is a topical anesthetic commonly used to temporarily relieve pain, itching, or swelling associated with anorectal disorders as well as relieve pain during blood draws in humans.^1^ For the research dogs in this case, the cream was used for pain relief during the blood draw and to prevent negative behavioral reactions during future blood draws. The amorphous component of the material seen on the peripheral blood smear is somewhat similar to what is found on cytology samples having ultrasound gel contamination.^2^ This may be due to ultrasound gel and the lidocaine cream having similar ingredients (disodium EDTA, distilled water, and propylene glycol). However, the lidocaine cream has a more globular appearance than the granular, bright purple material associated with ultrasound gel.

This article highlights the importance of knowing the characteristics of artifacts that can be seen in peripheral blood smears of animals. Different artifacts, such as lidocaine cream and ultrasound gel, will have different appearances on blood smears stained with Modified Wright stain, and knowing these characteristics will allow pathologists and technicians to identify these artifacts and relay findings to the clinicians.

In conclusion, the globular to amorphous, purple to magenta material seen on the peripheral blood smears of the research dogs was due to lidocaine cream contamination of the blood sample during collection. The addition of lidocaine cream to blood samples, ex vivo, supports this conclusion. To the authors' knowledge, documentation of this finding has not been previously reported.

## CONFLICT OF INTEREST STATEMENT

The authors have no conflicts of interest.
